# Impact and Efficacy of Topical Dry Mouth Products in Haemodialysis Patients with Xerostomia: A Pilot Study

**DOI:** 10.3290/j.ohpd.b871063

**Published:** 2020-12-14

**Authors:** Rosa María López-Pintor, Lucía López-Pintor, José González-Serrano, Elisabeth Casañas, Lorenzo de Arriba, Gonzalo Hernández

**Affiliations:** a Associate Professor, Department of Dental Clinical Specialties, ORALMED Research Group, School of Dentistry, Complutense University, Madrid, Spain. Study design and concept; instructed the patients; wrote the paper.; b Nursing Graduate, Hospital General La Mancha Centro, Alcázar de San Juan, Ciudad Real, Spain. Patient recruitment and data collection.; c Researcher, Department of Dental Clinical Specialties, ORALMED Research Group, School of Dentistry, Complutense University, Madrid, Spain. Write-up and editing of the paper.; d Researcher, Department of Dental Clinical Specialties, ORALMED Research Group, School of Dentistry, Complutense University, Madrid, Spain. Data analysis; write-up and editing of the paper.; e Associate Professor, Department of Dental Clinical Specialties, ORALMED Research Group, School of Dentistry, Complutense University, Madrid, Spain. Critically revised the manuscript for important intellectual content.; f Professor and Chair of Oral Medicine, Department of Dental Clinical Specialties, ORALMED Reserach Group, School of Dentistry, Complutense University, Madrid, Spain. Critically revised the manuscript for important intellectual content.

**Keywords:** haemodialysis patients, xerostomia treatment, topical treatment, interdialytic weight gain, oral health-related quality of life

## Abstract

**Purpose:**

Previous studies have shown that haemodialysis (HD) patients frequently suffer from xerostomia. This problem is associated with difficulties in oral functions, increased risk of oral diseases and interdialytic weight gain (IDWG). The aims of this preliminary study are to evaluate the efficacy and safety of topical dry mouth products in treating xerostomia in HD patients and their impact in reducing IDWG.

**Materials and Methods:**

We included 25 HD patients that suffered from xerostomia and complied with inclusion criteria. Subjects received toothpaste, mouthwash and mouth gel for dry mouth. The duration of treatment was 4 weeks. Patients filled out a visual analogue scale (VAS) for xerostomia and Oral Heath Impact Profile (OHIP)-14 questionnaires. Clinical data and IDWG were collected of the medical history of HD patients. Questionnaires and IDWG were collected at baseline, 2 and 4 weeks post-treatment.

**Results:**

Twenty-five patients completed the study (56% male and 44% female). The mean age was 63.52 ± 12.50 years. The topical treatment statistically significantly (p = 0.0001) alleviated the symptoms of xerostomia and improved their quality of life (OHIP-14 scores) (p = 0.0001). We observed a statistically significantly IDWG (kg) and IDWG% reduction (p = 0.03) after the use of topical treatment for xerostomia. None of the patients reported side effects.

**Conclusions:**

Xerostomia might affect the quality of life and increase the IDWG of these patients. The daily use of topical treatment for xerostomia could decrease thirst and IDWG, improving the quality of life of HD patients. Future randomised studies are needed to confirm these results.

There are more than two million people worldwide that suffer end-stage renal disease (ESRD). About 81,373 individuals commenced renal replacement therapy for ESRD in Europe in 2015, according to the European Renal Association-European Dialysis and Transplant Association Registry. Many of these people are receiving haemodialysis (HD) and a small number of them are treated with home dialysis or renal transplant.^17^,^19^

HD patients frequently suffer from oral diseases including xerostomia, oral mucosal lesions like petechiae, oral infections, adverse effects related to drug therapy such as gingival hyperplasia, dental anomalies and bone lesions.^8^,^16^,^34^ Xerostomia is a frequent problem in HD patients and its prevalence ranges from 32 to 81%.^6^,^7^,^11^,^14^,^16^,^21^,^22^,^29^,^33^ The causes of xerostomia in HD patients are not entirely known but there are different factors that may favour its appearance, such as old age, medication use, fluid intake restriction, reduced salivary flow, minor salivary glands parenchymal fibrosis and atrophy, mouth breathing, stress and anxiety.^4^,^7^,^14^,^15^,^27^ Xerostomia makes it difficult to speak, taste, swallow and chew properly, and increases the risk of oral diseases.^4^,^12^,^29^

Interdialytic weight gain (IDWG) is an important parameter in HD. It is assessed at the beginning of each HD session which along with clinical symptoms and signs and predialysis blood pressure. IDWG is necessary to make decisions regarding the amount of fluid removal during a HD session.^30^ There are studies that have correlated xerostomia to a higher IDWG.^4^,^6^,^10^ Patients with dry mouth increase their fluid intake, increasing at the same time IDWG. Non-compliance to the fluid restriction with HD patients produces fluid overload that increases cardiovascular problems like hypertension and pulmonary oedema, decreasing quality of life.^4^

Previous studies have evaluated the treatment of xerostomia in HD patients. Different treatments have been used to treat xerostomia in these patients: chewing gum,^5^,^9^,^13^ artificial saliva,^5^ liquorice mouthwash,^36^ pilocarpine,^32^ psychological intervention^1^ and acupressure.^35^ Some of these studies have found positive results in decreasing xerostomia,^5^,^32^,^35^,^36^ and only one study has observed a reduction in IDWG.^32^ No study has evaluated the improvement of quality of life after xerostomia treatment in these patients.

There are previous studies that showed the effectiveness of topical products for xerostomia containing aloe vera, betaine, allantoin, sodium fluoride and/or xylitol in patients suffering dry mouth.^3^,^12^,^23^,^25^,^31^ These active ingredients provide many benefits for treating xerostomia and its side effects. Aloe vera moisturises and helps to protect the sensitive oral mucosa. Betaine maintains humidity protecting from irritations. Allantoin accelerates wound healing. And finally, xylitol and sodium fluoride both have an anticaries effect.

No studies have evaluated xerostomia treatment with topical products containing betaine, allantoin, aloe vera, fluoride and xylitol in HD patients suffering from dry mouth. Therefore, the aim of this preliminary study is to evaluate the efficacy of topical dry mouth products (toothpaste, mouthwash and mouth gel) with the previous active ingredients in treating xerostomia in HD patients. We also want to assess the possible changes in their quality of life related to oral health and in their IDWG parameters. The working hypothesis is that the xerostomia treatment with these products could decrease xerostomia, reducing IDWG and improving quality of life.

## MATERIAL AND METHODS

### Study Design

We performed a pilot study trial to obtain preliminary evidence of the efficacy of topical dry mouth products in HD patients. The study was conducted in compliance with the principles of the Declaration of Helsinki. An ethics committee at Hospital San Carlos, Madrid, Spain, approved the study protocol (CI 15/101-E). An informed consent was obtained from each participant prior to his/her inclusion in the study.

### Participants

Patients included in this study received their HD treatment at the Asyter haemodialysis clinic in Alcázar de San Juan, Spain. All patients received a fixed HD schedule consisting of three times a week. The study was performed in the same clinic where HD patients attended for their HD treatment. Patients were consecutively invited to participate. The inclusion criteria were as follows: (a) patients suffering from xerostomia; (b) ≥ 4 month on HD; (c) ≥ 18 years of age; and (d) mentally and physically able to participate. Patients with haemodynamic instability preventing sufficient ultrafiltration, hospitalisation within the preceding 2 months, dementia or terminal diseases, logistic impossibility of investigation, using any medication or product for his/her dry mouth condition, and/or unwilling to participate in this study were excluded.

A dedicated HD nurse clinician (Lucía López-Pintor) collected the following variables at baseline from the clinic database: gender, age, time on HD, causes of ESRD, underlying diseases, consumed drugs, body mass index, dry weight, IDWG, tobacco and alcohol. Rosa María López-Pintor collected the variable ‘presence of dentures’.

Patients were recruited for this study from March 2015 to June 2015. Patients received treatment and were followed between September 2015 and December 2015.

### Interventions and Instructions to Patients

The patients included in this study received topical dry mouth products: toothpaste, mouth rinse and mouth gel provided by the company Dentaid S.L. These topical dry mouth products (Xeros Dentaid, Cerdanyola, Barcelona, Spain) contain: (a) toothpaste: betaine (4%), xylitol (10%), allantoin (0.10%), and sodium fluoride (0.321%, 1450 ppm); (b) mouth rinse: betaine (1.33%), xylitol (10%), allantoin (0.10%), and sodium fluoride (0.05%, 226 ppm); (c) mouth gel: betaine (1%), xylitol (10%), aloe vera (0.05%), and sodium fluoride (0.0033%, 15 ppm).

The participants were given instructions by Rosa María López-Pintor to brush their teeth three times a day with 0.5 g of toothpaste following the Bass brushing technique for 3 min and to rinse with 15 ml of mouthwash after each brushing for 30 s. They could not eat or drink for at least 30 min after using these products. We gave all the patients the same soft dental brush (Vitis Suave, Dentaid, Cedanyola, Barcelona, Spain) to brush their teeth. The mouth gel could be used topically, extending it through the gums, tongue, and cheeks (without rinsing) as many times as necessary, and especially before going to sleep. Patients had to use these products for 4 weeks (28 days). Patients were informed not to use other oral hygiene or xerostomia products apart from those used in this study.

### Assessment of IDWG

The trained nurse (Lucía López-Pintor) collected each patient’s weight before and after each dialysis session at baseline, 2 and 4 weeks post-treatment. IDWG was defined as the amount of fluid (kg) removed during the dialysis session. Dividing IDWG by the patient’s target dry weight and multiplying this result per 100, we obtained the percentage of IDWG. The target dry weight of the patient was determined according to the standard clinical criteria by nephrologists.

### Xerostomia

Patients who replied positively to the question: ‘Are you normally aware of your dry mouth?’ were considered to have xerostomia.^2^

### Xerostomia Visual Analogue Scale (VAS) Questionnaire

Patients filled out a VAS questionnaire about their dry mouth sensation. The questionnaire contains eight items addressing oral dryness: (1) Rate the difficulty you experience in speaking because of dryness; (2) Rate the difficulty you experience in swallowing because of dryness; (3) Rate how much saliva is in your mouth; (4) Rate the dryness of your mouth; (5) Rate the dryness of your throat; (6) Rate the dryness of your lips; (7) Rate the dryness of your tongue; and (8) Rate the level of your thirst.^26^ Each item could obtain values between 0–10 cm. Therefore, total scores ranged from 0 to 80 cm, where higher scores showed a direct relation to a higher xerostomia sensation. The VAS questionnaire was assessed before each dialysis session at baseline, and 2 and 4 weeks post-treatment. The VAS questionnaire was assessed before each dialysis session at baseline, and 2 and 4 weeks post-treatment.

### Assessment of Oral Health Impact Profile (OHIP)-14

The Spanish validated version of the OHIP-14 questionnaire was used to assess patient quality of life.^24^ The questionnaire consists of 14 items that explore different aspects of oral function and quality of life. Responses to this scale are based on a Likert format, with a five-point ordinal scale ranging from ‘never’ (coded 1) to ‘very often’ (coded 5). The score ranges from 14 to 70, where higher scores correspond to poorer oral quality of life.^20^ It was completed before each dialysis session at baseline, and 2 and 4 weeks post-treatment.

### Sample Size

We calculated the sample size considering that at least 40% of the HD patients would perceive any effect as an improvement to their dry mouth sensation. With a = 0.05, a statistical power of 90% to detect this difference would require at least 25 subjects in the study group.

### Statistical Analysis

The SPSS 22.0 statistical package (SPSS, Chicago, IL, USA) for Microsoft Windows was used for the statistical analysis of the results. A general descriptive analysis of each patient’s characteristics was made. We applied the Wilcoxon signed-rank test to compare two quantitative related samples or repeated measures. The Friedman test was used to compare three quantitative related samples. Differences were considered statistically significant if p was less or equal than 0.05.

### Results

Of the 50 patients who were old enough and met the medical and psychological criteria for participation, 22 did not suffer xerostomia, 1 was using other dry mouth products and 2 did not want to participate. Finally, we included 25 HD patients that suffered from xerostomia and complied with the inclusion criteria. We included 14 men and 11 women, mean age 63.52 ± 12.50 years. These patients were receiving HD treatment for 49.08 ± 54.43 months. The patients’ clinical characteristics can be observed in Table 1.

**Table 1:** Characteristics of HD patients (n = 25) of the study

Variables	n (%) or mean ± SD
Gender Male Female	14 (56%) 11 (44%)
Age (years)	63.52 ± 12.50
Time on HD (months)	49.08 ± 54.43
Causes of ESRD Chronic glomerulonephritis Polycystic kidney disease Diabetic nephropathy Pyelonephritis Others	8 (32%) 3 (12%) 2 (8%) 3 (12%) 9 (36%)
Diabetes Type 1 Type 2	2 (8%) 3 (12%)
Hypertension	14 (56%)
Body mass index	28.92 ± 5.52
Dry weight (kg)	76.42 ± 15.13
IDWG average (kg)	2.05 ± 1.11
Smokers	0 (0%)
Alcohol	0 (0%)
Dentures	11 (44%)

Values are number (%) and mean ± standard deviation (SD).

**Fig 1:**
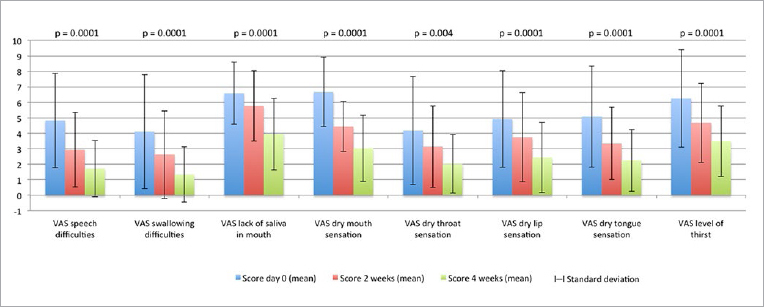
Evolution of the VAS for xerostomia during the study.

We observed that 80% and 92% of the HD patients showed improvement with xerostomia at the end of 2 and 4 weeks, respectively. Total xerostomia scores decreased progressively at 2 and 4 weeks after treatment (baseline 42.64 ± 14.64; 2 weeks 30.68 ± 11.12; 4 weeks 20.22 ± 8.35). In Figure 1 we present the improvement in each VAS item for xerostomia. Statistically significant improvements were seen in all items and total VAS score within 2 and 4 weeks (p = 0.0001) after treatment. VAS scores were lower at 4 weeks than 2 weeks after treatment.

The results of OHIP-14 scores are presented on Table 2. The quality of life of these patients improved statistically significantly (p = 0.0001) after treatment. In the same way as for VAS, every OHIP-14 item result was lower at 4 weeks vs 2 weeks.

**Table 2:** OHIP-14 scores through treatment

OHIP-14	Score day 0 (mean ± SD)	Score 2 weeks (mean ± SD)	Score 4 weeks (mean ± SD)	*p* value
Functional limitation Problems pronouncing correctly Unpleasant taste sensation	5.36 ± 2.56 2.80 ± 1.44 2.56 ± 1.56	4.04 ± 1.81 2.28 ± 1.20 1.76 ± 0.97	2.80 ± 1.04 1.56 ± 0.92 1.24 ± 0.44	0.0001 0.0001 0.0001
Physical pain Discomfort or pain Discomfort while eating	4.68 ± 2.46 2.12 ± 1.42 2.56 ± 1.61	3.60 ± 1.53 1.84 ± 1.11 1.76 ± 1.01	2.40 ± 0.76 1.16 ± 0.47 1.24 ± 0.52	0.0001 0.0001 0.0001
Psychological discomfort Shyness Concern	4.88 ± 2.20 2.64 ± 1.29 2.24 ± 1.30	4.04 ± 2.42 2.32 ± 1.41 1.72 ± 1.27	2.72 ± 1.43 1.48 ± 0.96 1.24 ± 0.66	0.0001 0.0001 0.001
Physical disability Eating dissatisfaction Interruption of meals	4.28 ± 2.57 2.28 ± 1.49 2.00 ± 1.32	3.08 ± 1.35 1.72 ± 1.06 1.36 ± 0.64	2.40 ± 0.96 1.28 ± 0.73 1.12 ± 0.44	0.0001 0.0001 0.001
Psychological disability Tension or anxiety Shame or pity	3.96 ± 1.92 2.08 ± 1.29 1.88 ± 1.27	2.72 ± 1.02 1.24 ± 0.44 1.48 ± 0.82	2.16 ± 0.47 1.00 ± 0.00 1.16 ± 0.47	0.0001 0.0001 0.001
Social disability Susceptibility/irritability Alteration in usual habits	3.60 ± 2.51 1.80 ± 1.26 1.80 ± 1.26	2.80 ± 1.68 1.48 ± 0.96 1.32 ± 0.80	2.24 ± 1.01 1.12 ± 0.60 1.12 ± 0.44	0.0001 0.001 0.0001
Handicap Scantly satisfactory life sensation Incapacity to lead a normal life	3.52 ± 1.87 2.00 ± 1.29 1.52 ± 0.87	2.76 ± 1.51 1.40 ± 0.76 1.36 ± 0.86	2.24 ± 0.88 1.12 ± 0.44 1.12 ± 0.44	0.0001 0.002 0.17
Total OHIP-14	30.28 ± 13.50	23.04 ± 8.93	16.96 ± 5.15	0.0001

OHIP-14, Oral Health Impact Profile-14; Friedman Test was applied.

IDWG (kg) and IDWG (%) decreased statistically significantly after 2 weeks of treatment. This reduction was maintained up to 4 weeks, observing statistically significant differences at baseline and 4 weeks after treatment (p = 0.03). We did not observe statistically significant differences between 2 and 4 weeks post-treatment (Table 3).

**Table 3:** Evolution of the Interdialytic Weight Gain

	Score day 0 (mean ± SD)	Score 2 weeks (mean ± SD)	Score 4 weeks (mean ± SD)	P^1^	P^2^	P^3^	P^4^
IDWG (kg)	2.05 ± 1.11	1.75 ± 0.91	1.71 ± 0.86	0.04	0.56	0.03	0.11
IDWG (%)	2.73 ± 1.44	2.31 ± 1.20	2.22 ± 1.05	0.05	0.54	0.03	0.11

P^1^, Wilcoxon test was applied, IDWG baseline-IDWG 2 weeks; P^2^, Wilcoxon test was applied, IDWG day 2-IDWG 4 weeks; P^3^, Wilcoxon test was applied, IDWG baseline-IDWG 4 weeks; P^4^, Friedman test was applied, IDWG baseline-IDWG 2 weeks-IDWG 4 weeks.

No side effects were found.

## Discussion

ESRD is a chronic disease that has an important negative impact in a patient’s quality of life. HD is a complex treatment that requires frequent visits to dialysis centres, usually three times a week. A great proportion of HD patients suffer from xerostomia that varies from 32 to 81%.^12^,^21^,^22^,^29^

As we mentioned earlier, the efficacy of different xerostomia treatments has been evaluated in HD patients. But at this time there is no strong evidence to be able to recommend a specific treatment. Sugar-free chewing gum is a mechanical saliva stimulator well tolerated by HD patients, but the results of different studies about this treatment are controversial.^5^,^9^,^13^ There are studies^24^,^32^ that obtained good results with pilocarpine reducing dry mouth sensation, and increasing salivary flow rate, but pilocarpine had important adverse effects. To avoid these secondary effects in medically involved patients like HD patients, we have other alternatives as palliative treatments including saliva substitutes and other therapies such as acupressure^35^ and psychological interventions.^1^

Palliative treatment includes saliva substitutes and the use of different oral health hygiene products for xerostomia. Bots et al^5^ completed a study using artificial saliva in HD patients that obtained a xerostomia reduction. Yu et al^36^ assessed the efficacy of a liquorice mouthwash to reduce xerostomia in HD. Liquorice improved dry mouth sensation, and increased salivary flow. In our study we observed a statistically significant improvement in xerostomia in HD patients using xerostomia products containing betaine, allantoin, aloe vera, fluoride and xylitol. At the end of the study, 92% of the patients obtained improvement, and the total VAS levels dropped by half. It is impossible to compare our results with other previous studies about xerostomia treatment in HD patients. Each previous study used a different questionnaire to evaluate xerostomia (VAS for thirst, one item VAS for xerostomia, dialysis thirst inventory and xerostomia inventory), did not show the percentage of patients who improved and treatment time was different. But all discussed studies showed that palliative treatment might be useful to treat HD xerostomia in HD patients, and had no side effects. In addition, the present topical xerostomia treatment helps to prevent other oral diseases such as caries and periodontal disease.

Oral health-related quality of life has been previously studied in HD patients.^18^,^22^,^28^ OHIP-14 is a validated questionnaire that measures patient’s functional limitation, discomfort and disability attributed to oral diseases and it is used worldwide.^24^ The results of this study show a great and significantly improvement in patient’s quality of life. OHIP-14 scores decreased 23% and 46.67% after 2 and 4 weeks, respectively. Therefore, treating xerostomia with products containing betaine, allantoin and aloe vera improves a HD patient’s quality of life, an important trait in these chronic patients.

Previous studies have shown a positive correlation between a higher IDWG and greater xerostomia sensation.^1^,^6^ HD patients have to follow restrictions on fluids and dietary salt intake to avoid an IDWG increase. Dry mouth increases thirst sensation that usually causes an increase of fluid intake. It is logical to think that xerostomia treatment may reduce IDWG. There are previous studies that evaluate the impact of xerostomia treatment in IDWG. Bots et al^5^ and Jagodzinska et al^13^ did not find a reduction of IDWG after xerostomia treatment with chewing gum. Sung et al^32^ showed a decreased of IDWG after the use of pilocarpine to treat dry mouth. Like Sung et al^32^, we found a statistical significance in IDWG reduction after 4 weeks of treatment with Xeros topical products. These results are promising, since this treatment could help to control the clinical condition of HD patients maintaining a proper volume balance.

This preliminary study has some limitations. One of them is the trial design, a pilot study, without a placebo group. As we know, placebo treatment could also have a positive effect in xerostomia. Therefore, this study was necessary to justify the cost of making similar placebo products in taste, colour and appearance and extending the sample for the conduct of a future randomised study. Another limitation of the study is that we cannot know the effect that each of the products (toothpaste, mouth rinse and mouth gel) has individually. Therefore, future work should compare whether the combined use of several products from the same line significantly influences the decrease in xerostomia. And another limitation is the short-term period of treatment (4 weeks). For this reason, the results of this study have to be confirmed with a future long-lasting randomised clinical trial. The greatest strengths of this study are the important efficacy obtained and the positive results in objective variables as IDWG reduction.

## Conclusion

In conclusion, treatment with xerostomia products containing betaine, allantoin and aloe vera reduced xerostomia and improve oral-related quality of life in HD patients reducing IDWG parameters. It is necessary to complete future randomised clinical trials testing these products vs a placebo over longer time periods to reassure these results.

## References

[ref1] Bellomo G, Coccetta P, Pasticci F, Rossi D, Selvi A (2015). The effect of psychological intervention on thirst and interdialytic weight gain in patients on chronic hemodialysis: a randomized controlled trial. J Ren Nutr.

[ref2] Bergdahl M, Bergdahl J (2000). Low unstimulated salivary flow and subjective oral dryness: association with medication, anxiety, depression, and stress. J Dent Res.

[ref3] Bin Mohsin AH, Reddy SV, Kumar MP, Samee S (2017). Aloe vera for dry mouth denture patients-Palliative therapy. J Clin Diag Res.

[ref4] Bossola M, Tazza L (2012). Xerostomia in patients on chronic hemodialysis. Nat Rev Nephrol.

[ref5] Bots CP, Brand HS, Veerman EC, Korevaar JC, Valentijn-Benz M, Bezemer PD (2005). Chewing gum and a saliva substitute alleviate thirst and xerostomia in patients on haemodialysis. Nephrol Dial Transplant.

[ref6] Bruzda-Zwiech A, Szczepanska J, Zwiech R (2014). Sodium gradient, xerostomia, thirst and inter-dialytic excessive weight gain: a possible relationship with hyposalivation in patients on maintenance hemodialysis. Int Urol Nephrol.

[ref7] de la Rosa Garcia E, Mondragon Padilla A, Aranda Romo S, Bustamante Ramirez MA (2006). Oral mucosa symptoms, signs and lesions, in end stage renal disease and non-end stage renal disease diabetic patients. Med Oral Patol Oral Cir Bucal.

[ref8] Dioguardi M, Caloro GA, Troiano G, Giannatempo G, Laino L, Petruzzi M (2015). Oral manifestations in chronic uremia patients. Ren Fail.

[ref9] Duruk N, Eser I (2016). The null effect of chewing gum during hemodialysis on dry mouth. Clin Nurse Spec.

[ref10] Fan WF, Zhang Q, Luo LH, Niu JY, Gu Y (2013). Study on the clinical significance and related factors of thirst and xerostomia in maintenance hemodialysis patients. Kidney Blood Press Res.

[ref11] Gavalda C, Bagan J, Scully C, Silvestre F, Milian M, Jimenez Y (1999). Renal hemodialysis patients: oral, salivary, dental and periodontal findings in 105 adult cases. Oral Dis.

[ref12] Gomez-Moreno G, Guardia J, Aguilar-Salvatierra A, Cabrera-Ayala M, Mate-Sanchez de-Val JE, Calvo-Guirado JL (2013). Effectiveness of malic acid 1% in patients with xerostomia induced by antihypertensive drugs. Med Oral Patol Oral Cir Bucal.

[ref13] Jagodzinska M, Zimmer-Nowicka J, Nowicki M (2011). Three months of regular gum chewing neither alleviates xerostomia nor reduces overhydration in chronic hemodialysis patients. J Ren Nut.

[ref14] Kao CH, Hsieh JF, Tsai SC, Ho YJ, Chang HR (2000). Decreased salivary function in patients with end-stage renal disease requiring hemodialysis. Am J Kidney.

[ref15] Kaya M CT, Ustun F, Sen S, Berkarda S (2002). Salivary function in patients with chronic renal failure undergoing hemodialysis. Ann Nucl Med.

[ref16] Klassen JT, Krasko BM (2002). The dental health status of dialysis patients. J Can Dent Assoc.

[ref17] Kramer A, Pippias M, Noordzij M, Stel VS, Afentakis N, Ambühl PM (2018). The European Renal Association-European Dialysis and Transplant Association (ERA-EDTA) Registry Annual Report 2015: a summary. Clin Kidney J.

[ref18] Lira E, Silva JA, Bernardino IM, da Silva JRC, Lima TLMA, Soares RSC, d’Avila S (2017). Quality of life related to oral health of patients undergoing hemodialysis and associated factors. Spec Care Dentist.

[ref19] Liyanage T, Ninomiya T, Jha V, Neal B, Hall MP, Okpechi I (2015). Worldwide access to treatment for end-stage kidney disease: a systematic review. Lancet.

[ref20] Locker D, Allen P (2002). Developing short-form measures of oral health-related quality of life. J Public Health Dent.

[ref21] Löfgren C, Wickström C, Sonesson M, Lagunas P, Christersson C (2012). A systematic review of methods to diagnose oral dryness and salivary gland function. BMC Oral Health.

[ref22] López-Pintor RM, López-Pintor L, Casañas E, de Arriba L, Hernández G (2017). Risk factors associated with xerostomia in haemodialysis patients. Med Oral Patol Oral Cir Bucal.

[ref23] Martín M, Marín A, López M, Liñán O, Alvarenga F, Büchser D (2017). Products based on olive oil, betaine, and xylitol in the post-radiotherapy xerostomia. Rep Pract Oncol Radiother.

[ref24] Montero-Martín J, Bravo-Pérez M, Albaladejo-Martínez A, Hernández-Martín L, Rosel-Gallardo E (2009). Validation of the Oral Health Impact Profile (OHIP-14sp) for adults in Spain. Med Oral Patol oral Cir Bucal.

[ref25] Morales-Bozo I, Rojas G, Ortega-Pinto A, Espinoza I, Soto L, Plaza A (2012). Evaluation of the efficacy of two mouthrinses formulated for the relief of xerostomia of diverse origin in adult subjects. Gerodontol.

[ref26] Pai S, Ghezzi E, Ship J (2001). Development of a Visual Analogue Scale questionnaire for subjective assessment of salivary dysfunction. Oral Sur Oral Med Oral Pathol Oral Radiol Endod.

[ref27] Postorino M, Catalano C, Martorano C, Cutrupi S, Marino C, Cozzupoli P (2003). Salivary and lacrimal secretion is reduced in patients with ESRD. Am J Kidney Dis.

[ref28] Rodakowska E, Wilczynska-Borawska M, Fryc J, Baginska J, Naumnik B (2018). Oral Health-related quality of life in patients undergoing chronic hemodialysis. Patient Prefer Adherence.

[ref29] Saleh J, Figueiredo MA, Cherubini K, Salum FG (2015). Salivary hypofunction: an update on aetiology, diagnosis and therapeutics. Arch Oral Biol.

[ref30] Sarkar SR, Kotanko P, Levin NW (2006). Interdialytic weight gain: implications in hemodialysis patients. Semin Dial.

[ref31] Ship J, McCutcheon J, Spivakovsky S, Ker AR (2007). Safety and effectiveness of topical dry mouth product containing olive oil, betaine, and xylitol in reducing xerostomia for polipharmacy-induced dry mouth. J Oral Rehabil.

[ref32] Sung JM, Kuo SC, Guo HR, Chuang SF, Lee SY, Huang JJ (2005). Decreased salivary flow rate as a dipsogenic factor in hemodialysis patients: evidence from an observational study and a pilocarpine clinical trial. J Am Soc Nephrol.

[ref33] Swapna LA, Reddy RS, Ramesh T, Reddy RL, Vijayalaxmi N, Karmakar P (2013). Oral health status in haemodialysis patients. J Clin Diagn Res.

[ref34] Teratani G, Awano S, Soh I, Yoshida A, Kinoshita N, Hamasaki T (2013). Oral health in patients on haemodialysis for diabetic nephropathy and chronic glomerulonephritis. Clin Oral Investig.

[ref35] Yang LY, Yates P, Chin CC, Kao TK (2010). Effect of acupressure on thirst in hemodialysis patients. Kidney Blood Press Res.

[ref36] Yu IC, Tsai YF, Fang JT, Yeh MM, Fang JY, Liu CY (2016). Effects of mouthwash interventions on xerostomia and unstimulated whole saliva flow rate among hemodialysis patients: a randomized controlled study. Int J Nurs Stud.

